# Plans for Nationwide Serosurveillance Network in Vietnam

**DOI:** 10.3201/eid2601.190641

**Published:** 2020-01

**Authors:** Dang Duc Anh, Marc Choisy, Hannah E. Clapham, Hoang Quoc Cuong, Vu Tien Viet Dung, Tran Nhu Duong, Nguyen Le Khanh Hang, Hoang Thi Thu Ha, Nguyen Tran Hien, Tran Thi Nguyen Hoa, Tran Thi Mai Hung, Vu Thi Lan Huong, Dang Thi Thanh Huyen, Nguyen Cong Khanh, Sonia O. Lewycka, Ezra Linley, Le Thi Quynh Mai, Behzad Nadjm, Ngu Duy Nghia, Richard Pebody, Hoang Vu Mai Phuong, Luong Minh Tan, Le Van Tan, Pham Quang Thai, Le Viet Thanh, Nguyen Thi Le Thanh, Nguyen Thi Thu Thuy, Nguyen Thi Thuong, Le Thị Thanh, Nguyen Thi Thanh Thao, Nguyen Anh Tuan, Phan Thi Ngoc Uyen, H. Rogier van Doorn

**Affiliations:** National Institute of Hygiene and Epidemiology, Hanoi, Vietnam (D.D. Anh, T.N. Duong, N.L.K. Hang, H.T.T. Ha, N.T. Hien, T.T.N. Hoa, T.T.M. Hung, D.T.T. Huyen, N.C. Khanh, L.T.Q. Mai, N.D. Nghia, H.V.M. Phuong, L.M. Tan, P.Q. Thai, N.T.T. Thuy, N.T. Thuong, L.T. Thanh, N.A. Tuan, H.R. van Doorn);; Institut de Recherche, Marseille, France (M. Choisy);; Oxford University Clinical Research Unit, Ho Chi Minh City and Hanoi, Vietnam (M. Choisy, H.E. Clapham, V.T.V. Dung, V.T.L. Huong, S.O. Lewycka, B. Nadjm, L.V. Tan, L.V. Thanh, N.T.L. Thanh);; University of Oxford, Oxford, UK (H.E. Clapham, S.O. Lewycka, B. Nadjm, H.R. van Doorn);; Pasteur Institute, Ho Chi Minh City (H.Q. Cuong, N.T.T. Thao, P.T.N. Uyen);; Public Health England, London, UK (E. Linley, R. Pebody)

**Keywords:** Serology, vaccine-preventable diseases, serosurveillance, seroepidemiology, Vietnam

## Abstract

In recent years, serosurveillance has gained momentum as a way of determining disease transmission and immunity in populations, particularly with respect to vaccine-preventable diseases. At the end of 2017, the Oxford University Clinical Research Unit and the National Institute of Hygiene and Epidemiology held a meeting in Vietnam with national policy makers, researchers, and international experts to discuss current seroepidemiologic projects in Vietnam and future needs and plans for nationwide serosurveillance. This report summarizes the meeting and the plans that were discussed to set up nationwide serosurveillance in Vietnam.

Seroepidemiology is the practice of measuring the serologic response to infectious diseases in a population to determine the epidemiology of a pathogen. The measured serologic responses can result from infection, vaccination, or both, or may have been acquired passively in very young children (maternal antibodies). Depending on the pathogen, these responses can provide information about transmission, immunity, and vaccination rates. Use of biomarkers and serology to estimate population immunity and disease transmission has increasingly gained interest (*1**–**4*).

The ideal way to collect serum from a population for seroepidemiology is to conduct population-representative serum sampling projects, such as those conducted recently for dengue in Bangladesh (*5*) and India (*6*). In addition, samples collected to test for certain pathogens can be extended to other pathogens, such as for parasitic diseases in Cambodia (*7*) and tetanus in Kenya, Tanzania, and Mozambique (*8*). However, in general, population-based surveys are expensive and time-consuming and therefore difficult to conduct regularly. An alternative, more convenient and less expensive way to collect serum samples is to use residual serum from laboratories. However, collection in this way will not necessarily be representative of the population. If serum collection is implemented continually over a long period (although not necessarily from the same persons), it can become a serosurveillance system. Many countries in Europe have a serosurveillance system that uses residual serum from hospital laboratories (*9*).

In the absence of reliable data on vaccination coverage, which is often the situation, a representative national serosurveillance system is a valuable tool for assessing population age-specific and region-specific immunity against vaccine-preventable diseases. This method has been applied in 18 countries in Europe to inform vaccine policies and measles and rubella elimination programs (*10*,*11*). This information can be used for evaluation of the effect and duration of protection from vaccines and for modeling and health economic evaluations of current or future vaccination programs. Currently, however, a defined role for serologic data in such programs is lacking, as highlighted by the Strategic Advisory Group of Experts on Immunization in 2019 (*12*). For countries like Vietnam, establishment of a national serosurveillance bank could be well-timed for monitoring vaccine program coverage because international funding for vaccine programs began to be withdrawn when Vietnam’s economic status transitioned from low to lower middle income in 2013. This transition will be relevant for ensuring ongoing high coverage of existing vaccinations (including issues of lack of access to vaccines and increasing vaccination refusal [*13**,**14*]) and for ascertaining the need and locations for introduction of new vaccines (*15*) and targeted booster campaigns. In addition, seroepidemiology can also help determine transmission dynamics or transmission extent for diseases for which little is known or for which surveillance is currently limited.

Although serosurveillance work has been undertaken fairly widely in Europe, less serosurveillance work has been conducted in other regions of the world, although it has been proposed as a useful tool in other regions (*4*). With the view of assessing the possibilities for serosurveillance in Vietnam, we reviewed what has been done in this country and how lessons from elsewhere could be applied, and we suggest steps to move forward with a serosurveillance network in Vietnam.

In 2017, the Oxford University Clinical Research Unit (OUCRU) Vietnam, in collaboration with the National Institute of Hygiene and Epidemiology (NIHE), organized a meeting to discuss the possibility of nationwide serosurveillance for Vietnam. Collaborators from Public Health England and the Pasteur Institute (Ho Chi Minh City) joined to share their expertise. The meeting was held in Hanoi, Vietnam, on October 16–17, 2017.

## Meeting Summary

Our aim with this meeting was to explore establishment of a representative national serosurveillance network for Vietnam to continue seroepidemiology work and to assess the need for, and how best to use, serosurveillance in policy making in Vietnam. We envisaged that this network would help assess coverage of vaccination programs and identify at-risk regions, model health and economic benefits of future vaccine implementation, and provide information about the presence and risk of novel pathogens.

At this meeting, we discussed the present design of and results from serum collections established in Vietnam by NIHE, OUCRU, and their partners and by Public Health England and its partners; public health questions relevant for Vietnam that such a network should be able to answer and availability of assays to address these questions; ways of collecting serum (hospital, outpatient clinics, community) and their benefits and biases and how to best understand and avoid these biases; and feasible ways to collect representative samples for national serosurveillance in Vietnam, making optimal use of existing serosurveillance programs (OUCRU) and demographic surveillance sites.

We present a summary of the discussions and the main outcomes of the meeting. This report provides a roadmap for the implementation of a nationally representative serosurveillance network.

## Types of Relevant Serosurveillance Collections already Undertaken and Scientific Uses

### Europe

The European Sero-Epidemiology Network was established across Europe in 1995 to compare the seroepidemiology of a range of key vaccine-preventable infections and provide vaccine recommendations across countries (*9*). Different countries had different collection methods; some were population based and others collected convenience samples. Tests were standardized across countries by testing of a reference panel, similar to external quality assessment panels for diagnostic laboratories (*16*).

The Public Health England residual serum bank receives samples from diagnostic laboratories around the country and stores these samples centrally. Public Health England has worked out standard operating procedures for receiving, storing, and sending these samples out for research purposes. This seroepidemiology collection has been ongoing since 1986, and the archive now contains ≈200,000 samples. This procedure provides an excellent example of how to streamline ongoing sample collection and use.

Serologic testing of these samples has been used to guide a range of existing and new vaccination programs. A particular example is provided by measles, mumps, and rubella, for which serologic studies, combined with mathematical modeling, led to catch-up vaccinations being offered to susceptible cohorts in the United Kingdom (*10*,*17*). Serologic testing has also been of use for updating diphtheria vaccination recommendation across Europe (*18*). Research on age-specific seroprevalence with regard to human papillomavirus, which used samples from this collection, formed part of the evidence to introduce the human papillomavirus vaccine in the United Kingdom (*19*). These samples have also been used to determine the extent of influenza transmission and background population immunity during the 2009 pandemic; samples from the serum bank were tested for antibodies before the pandemic and after the 2 waves of transmission (*20*).

### Vietnam

For some time, serologic surveys and serosurveillance have been of interest to public health agencies and research institutes across Vietnam. In some instances, studies have been conducted to research specific diseases; in other instances, samples were collected for more general use. The Institute Pasteur has undertaken multiple serum collections, which include studies of HIV, funded by World Bank or the World Health Organization, and other studies undertaken as part of vaccine trials. A recent example was a Zika virus serosurvey that used samples collected at nutrition centers in Ho Chi Minh City, Vietnam (participants <18 years of age), and at blood donation centers (participants <60 years of age).

The General Department of Preventive Medicine, Ministry of Health, NIHE, and 3 regional institutes are undertaking population representative sample collection from adults (>18 years of age) around the country; samples will be tested for hepatitis B and C viruses. The Expanded Programme on Immunization will conduct a similar seroprevalence survey of those <18 years of age. The sampling defined socioecologic (*21*) regions in Vietnam and randomly selected districts and communes, from which households were randomly selected. Sampling included urban and nonurban areas as well as persons from different ethnic groups. The samples are stored and can now be tested for antibodies to other diseases. Other recent work used serology to show a rapid decline in immunity to polio virus 2 after this vaccine was not used for 2 years (*22*).

NIHE has also undertaken targeted serosurveillance studies. An example is testing poultry workers for antibodies to influenza A(H5N1) virus, the results of which suggested low levels of transmission (*23*).

Since 2009, OUCRU has been conducting a serial seroepidemiology study in 13 provinces across central and southern Vietnam. This study collects residual serum from 200 persons of all ages, every 2–4 months, from 10 hospitals around the country (*24*). This study was started to study influenza, but the scope has since expanded. To date, 75,000 samples have been collected as part of this study, and this number continues to grow. In 2012–2013, OUCRU and NIHE collaborated to collect residual serum samples from 4 hospitals around the country; this study collected 1,300 residual samples from patients stratified by age (*14*,*25*). These samples have since been tested for antibodies to strongyloides (and showed high seroprevalence) (*25*) and to measles virus. Work from OUCRU assessed population exposure and immunity to chikungunya virus over time at 4 locations across Vietnam (*26*). Cohort studies in Vietnam have collected regular serum samples; these studies include the OUCRU birth cohort study (*27*,*28*), Ha Nam household cohort study (*29*), and NIHE-Nagasaki University cohort study in Nha Trang (*30*).

To estimate measles vaccination coverage across Vietnam, the NIHE/OUCRU samples from 2012–2013 have been tested for measles IgG. Test results were found to be a more reliable predictor of where gaps in immunity led to outbreaks in 2014 than were national coverage data or data from the UNICEF Multiple Indicator Cluster Surveys or Demographic and Health Surveys (*14*). Similarly, samples from the OUCRU 13-site serum bank have been tested for tetanus antibodies in Ho Chi Minh City; the results showed gaps in immunity to tetanus, consistent with the observed age distribution of tetanus case-patients, which should be addressed by additional vaccination (*31*).

## Potential Uses for Serosurveillance in Vietnam

Meeting participants decided that the main focuses of serosurveillance in Vietnam would be to test for antibodies to pathogens for which concerns exist about vaccination uptake or current schedules (e.g., measles, pertussis, tetanus, and diphtheria); vaccines may potentially be introduced in the future (e.g., enterovirus A71, influenza virus, and pneumococcal infection); and the extent of transmission is currently unknown or not fully quantified in Vietnam (e.g., Zika virus, avian influenza virus, or other emerging pathogens). For all of these considerations, use of serosurveillance relies on there being commercially available assays or availability of easily implementable, interpretable, and replicable in-house assays. For many vaccine-preventable diseases (e.g., measles or tetanus), these assays have been developed and are widely used, although interpretation may still be complicated (*32*). For other pathogens (e.g., enterovirus A71 or *Bordetella pertussis*), careful thought must be given to the assays used.

## Collection Types and Their Pros and Cons

For setting up serosurveillance or serologic studies, sampling techniques are debated. In general, collections can be done in 2 ways: random population-based sampling (e.g., the NIHE hepatitis B and C studies) and convenience sampling (e.g., the OUCRU serum banks). Random population-based collection has the advantage of being more representative of the population being sampled; however, it is very expensive and labor-intensive and therefore cannot be conducted regularly. Convenience sampling introduces bias because the sampled population may not be representative. However, this type of sampling is less expensive and easier to undertake and therefore can be conducted more regularly and may still provide relevant information, as has been illustrated for measles and tetanus in Vietnam (*14*,*31*).

## Vision for a Serosurveillance Network in Vietnam

Because of the pros and cons of each sampling approach, meeting participants decided to use a hybrid approach in Vietnam, extending previous studies undertaken in the country and learning from lessons in other countries. The hybrid approach will help clarify the biases in the convenience sampling methods, some of which may be specific to Vietnam. These biases include health-seeking behaviors and their association with factors such as socioeconomic status, ethnic group, and vaccination uptake. The locations will be chosen across the country to be as representative as possible, which is particularly necessary in a country with wide geographic and demographic variation.

## The Network and What Information to Collect

The backbone of the network was envisioned to consist of hospital-based residual serum collections with the following characteristics, extending and bringing together the 2 existing OUCRU studies in Vietnam. For the hospital-based collection of residual serum, we envisage collecting serum, stratified by patient age, from 20–25 centers. We intend to have age-stratified collection of serum with 1-year bands for patients 1–20 years of age and 5-year bands for patients 20–70 years of age, collecting a total of 200 samples every 3 months and storing them at −20°C, as has been done in previous studies (*14*,*25*). This method will ensure that collection is in line with the standards of the European Sero-Epidemiology Network (*16*). The centers (2–4 centers per region) will represent the 8 ecologic regions in Vietnam. These samples will be stored in Vietnam, with the possibility of sample sharing if approved by NIHE. This study will be submitted for approval by the NIHE, Oxford Tropical Research Ethics Committee, and OUCRU institutional review boards for testing for antibodies to pathogens relevant to Vietnam and with waived consent, as in previous studies in Europe (*9*). The serum bank will be funded on a per-project basis, but a business plan will be developed for national support through NIHE moving forward.

In tandem with setting up this hospital-based network, we will compare it with population-based studies already conducted by NIHE in Vietnam. This comparison will enable us to assess the biases of this type of collection by testing samples from the same time and location from both hospital-based and population-based serum collections concurrently for antibodies to the same antigen. The results will then be compared across patient ages and locations to look for areas where results are discordant, and we will attempt to summarize associated factors. Possible antigens for this testing include those against *C. tetani* and measles, hepatitis B, and hepatis C viruses. This comparison could be of interest to other countries considering the different ways to set up serum collection. We will also continue to assess any possibility of adding serum collection and testing to other data collection efforts.

For all testing of samples, we will work with Public Health England and other national reference institutes to choose sample storage, platforms, and assays that can be implemented in Vietnam for high throughput multiplex analysis of locally relevant diseases. In past work on this serum bank in Vietnam, individual ELISA-type tests have been used for the vaccine-preventable diseases (*14*), and multiplex assays have been used within pathogen groups (*24*). However, moving forward, to decide what best to use, we will explore use of multiplex assays (such as a Luminex assay) across multiple pathogens, considering cost, sample volume used, laboratory equipment required, ease of standardization, and information gained.

The results of studies of this serum bank will be communicated in scientific publications and in regular discussion with NIHE and the Vietnam Ministry of Health. OUCRU will tailor its public engagement program on vaccination to results of this project. 

We believe that serosurveillance could form an integral part of Vietnam’s future surveillance strategy for infectious diseases, particularly for vaccine-preventable diseases and emerging infections. Such systems have been of great use globally. In Vietnam, we are able to build on the current strengths of research and public health bodies to develop a nationwide surveillance system. Creating this system within existing public health bodies would additionally benefit from preestablished and functional relationships with policy makers. This system will be a vital tool for Vietnam in the next era of infectious disease control.
